# INTERGENERATIONAL RELATIONSHIPS AND HAPPINESS: MEDIATING ROLES OF PSYCHOLOGICAL RESILIENCE AND COVID-19 ANXIETY

**DOI:** 10.1093/geroni/igad104.0619

**Published:** 2023-12-21

**Authors:** Xue Bai, Shuai Zhou, Chi-Ko Lee

**Affiliations:** The Hong Kong Polytechnic University, Hong Kong, Hong Kong; The Hong Kong Polytechnic University, Hong Kong, Hong Kong; The Hong Kong Polytechnic University, Hong Kong, Hong Kong

## Abstract

The COVID-19 pandemic has largely worsened the well-being of older adults worldwide. Despite recent literature highlighting either social connectedness or psychological responses, whether and how intergenerational relationships influence the emotional well-being of older adults during the pandemic remains unclear. This study aimed to examine the association between intergenerational relationships and happiness among ageing Chinese adults during the pandemic and the mediating roles of psychological resilience and COVID-19 anxiety. A representative survey was conducted from November 2020-January 2021 to collect data from 819 Chinese adults aged 55 or above (52.4% females) in Hong Kong. Path models were used to examine the associations between intergenerational relationships, resilience, COVID-19 anxiety, and happiness. Regression results showed that intergenerational relationship quality was associated with more psychological resilience (b = 0.21, p < 0.001), lower COVID-19 anxiety (b = -0.14, p < 0.001), and a high level of happiness (b = 0.15, p < 0.001). COVID-19 anxiety partially mediated the effects of intergenerational relationship quality on happiness. Moreover, psychological resilience was associated with a higher level of happiness through ameliorated COVID-19 anxiety. This study elucidates that intergenerational relationship quality was positively associated with happiness during the pandemic and the association could be partly explained by enhanced psychological resilience and alleviated COVID-19 anxiety. The findings provide implications that targeted policies and services that strengthen intergenerational solidarity and psychological resilience may be effective in improving emotional well-being in older populations.

